# Supplementary material for: The effect of ethanol concentration on the morphological and molecular preservation of insects for biodiversity studies
Source: PeerJ. 2021 Feb 12;9:e10799. doi: 10.7717/peerj.10799 (PMC7883690; doi:10.7717/peerj.10799)
Supplement: Supplemental Information 1 — Methods for spike-in plasmid relative abundance quantification and bioinformatic processing of the sequencing data. Tables S1–S4, significance tables of the statistical analysis of the appendages experiments. [file peerj-09-10799-s001.pdf]

The effect of ethanol concentration on the morphological and molecular preservation of insects for biodiversity studies

Daniel Marquina, Mateusz Buczek, Fredrik Ronquist & Piotr Łukasik

Supplementary Material

Table Of Contents:

Quantification - spike-in plasmid 1
Amplicon data analysis pipeline 2
Table S1. 3
Table S2. 4
Table S3. 5
Table S4. 5

Quantification - spike-in plasmid

The insert sequence was developed based on *Calliophora vomitoria* COI sequence, and synthesized and inserted into vector backbone pEX-A128 by Eurofins Genomics. The plasmid was cloned into JO-FI competent cells derived from the E. coli DH5α strain (A&A Biotechnology) using CloneJET PCR Clonning kit (Thermo Fisher Scientific). A single successfully transformed colony was transferred into LB medium (A&A Biotechnology) mixed with Ampicilin Sodium (A&A Biotechnology) at the final concentration 100 ug/ml and grown overnight at 37˚C. Plasmids were extracted from an overnight culture using Plasmid Miniprep DNA Purification Kit (EurX), linearized using single-cutting enzyme AatII (New England Biosciences), quantified using Qubit dsDNA HS Assay Kit (Thermo Fisher Scientific), and diluted with TE to the working concentration.

The insert sequence, with BF3/BR2 primer sites underlined:

>COI_CallioSynth
GGAGCCCCAGATATAGCATTCCCTCGAATAAAATTATTACATATATTTGCTATACTTAGTTCACTATCGTAA
GCATAAATATACAGAATATGAAATATTGGAGCTGGAACTGGATGAACTGTTTATCCACCTTTATCGCCATGC
ATAACTCTTCACTGATCTAAGGACGATATGGTTAGTATCTGTATACTTAGACGATGTATAAAGTCGAATCAA
TGCGTTAGAAACATTTATAGAATAGATAATTAGACATATTGTACGCCTAATAAGAACAAGATAAGACGCTA
AATCCAATCAATTCTTCAAGTAGTATACTATACTGTACTATATTCTTTACCAGTATTAGCAGGAGCTATTACT
ATATTTCGATTACCTCTAGTTATAATAAATGTAATGATAAACGAAGTAATACAAGTAGAACTATTCATATAA
CATGATTTATTTTGATTTTTTGGTCATCCTGAAGTTTAT

Amplicon data analysis pipeline

The amplicon data were analyzed using Mothur v.1.44.2, using a custom pipeline. Commands are provided below.

```
   # Merging reads into contigs
make.contigs(file=DNA.txt)

   # variable-length insert primer trimming, screening contigs based on
   quality and length
trim.seqs(fasta=DNA.trim.contigs.fasta,
oligos=/mnt/matrix/symbio/db/references/primers_to_trim_VarLenIns.oligos,
processors=32, minlength=400, maxlength=430, maxambig=0, maxhomop=10,
pdiffs=2)
   # primers_to_trim_VarLenIns.oligos ---
   # oligos used for library prep:
   # COIBF3_P5   ACACTCTTTCCCTACACGACGCTCTTCCGATCT---
     {insert}CCHGAYATRGCHTTYCCHCG
   # COIBR2_P7   GTGACTGGAGTTCAGACGTGTGCTCTTCCGATCT---
     {insert)CDGGRTGNCCRAARAAYCA

   forward CCHGAYATRGCHTTYCCHCG
   forward ACCHGAYATRGCHTTYCCHCG
   forward GACCHGAYATRGCHTTYCCHCG
   forward TGACCHGAYATRGCHTTYCCHCG
   reverse CDGGRTGNCCRAARAAYCA
   reverse TCDGGRTGNCCRAARAAYCA
   reverse ATCDGGRTGNCCRAARAAYCA
   reverse GATCDGGRTGNCCRAARAAYCA

   # selecting only the sequences that survived the screen from the group
file
list.seqs(fasta=DNA.trim.contigs.trim.fasta)
get.seqs(accnos=DNA.trim.contigs.trim.accnos, group=DNA.contigs.groups)

   # read dereplication
unique.seqs(fasta=DNA.trim.contigs.trim.fasta)
count.seqs(name=DNA.trim.contigs.trim.names,
group=DNA.contigs.pick.groups, compress=f)

   # discarding singletons
split.abund(fasta=DNA.trim.contigs.trim.unique.fasta,
count=DNA.trim.contigs.trim.count_table, cutoff=1)

   # aligning reads against a custom reference - alignment of previously
   obtained COI sequences of the experimental species, the spike-in
   standard, and alphaproteobacterial symbionts
align.seqs(fasta=DNA.trim.contigs.trim.unique.abund.fasta,
reference=/mnt/matrix/symbio/db/references/COI_ref_seqs_fragility.fasta,
processors=40)
```

```
   # discarding unaligned or poorly aligned sequences, filtering the
   alignment
screen.seqs(fasta=DNA.trim.contigs.trim.unique.abund.align,
count=DNA.trim.contigs.trim.abund.count_table,         minlength=410,
maxlength=425, start=2, end=430)
filter.seqs(fasta=DNA.trim.contigs.trim.unique.abund.good.align,vertical=T
, trump=.)

   # clustering, using the Vsearch agc algorithm
cluster(fasta=DNA_final.fasta,  count=DNA_final.count_table,  method=agc,
cutoff=0.03)

   # based on clustering results, binning sequences, generating OTU table,
   and identifying representative sequences for OTUs
bin.seqs(list=DNA_final.agc.list, fasta=DNA_final.fasta, label=0.03)
make.shared(list=DNA_final.agc.list,        count=DNA_final.count_table,
label=0.03)
get.oturep(list=DNA_final.agc.list,       fasta=DNA_final.agc.0.03.fasta,
count=DNA_final.count_table, method=abundance, cutoff=0.03)
```

Table S1.

Species included in the communities, number of individuals used, expected brittleness, and origin.

Species	No. individuals	Brittleness	Origin
Macrolophus pygmaeus	10	Weak	Commercial purchase: Lindesro AB (Sweden)
Aphidoletes aphidimyza	10	Very weak	Commercial purchase: Lindesro AB (Sweden)
Drosophila hydei	10	Tough	Commercial purchase: Fibe AB (Sweden)
Dacnusa sibirica	10	Tough	Commercial purchase: Lindesro AB (Sweden)
Calliphora vomitoria	2	Intermediate	Commercial purchase: Reptilgrottan (Sweden)
Formica rufa	2	Very tough	Collected from NRM surroundings
Dermestes haemorrhoidalis	2	Very tough	Donated from NRM vertebrate collection

Table S2.

Pairwise comparisons for treatments with significant differences in Experiment 1. Treatment 1 is different from Treatment 2, being the mean number of appendages lost higher/lower in Treatment 2. Number = ethanol concentration, G = Gentle shaking, V= Violent shaking.

Species	Treatment 1	Treatment 2	Lower / Higher
Macrolophus pygameus	30G	80G, 90G, 95G, 97G	Higher
	30G	70V, 90V, 95V, 97V, 99V	Higher
	50G	95V	Higher
	95G	30V	Lower
	30V	95V	Higher
	50V	95V	Higher
	80V	95V	Higher
Aphidoletes aphidimyza	30G	90G, 95G, 97G, 99G	Higher
	30G	90V, 95V, 97V, 99V	Higher
	50G	90G, 95G, 97G, 99G	Higher
	50G	90V, 95V, 97V, 99V	Higher
	50G	30V	Higher
	70G	90G, 95G, 97G, 99G	Higher
	70G	30V	Higher
	70G	90V, 95V, 97V, 99V	Higher
	80G	90G, 95G, 97G, 99G	Higher
	80G	95V, 95V, 99V	Higher
	90G	30V, 50V, 70V, 80V	Lower
	90G	99V	Higher
	95G	30V, 50V, 70V, 80V	Lower
	97G	30V, 50V, 70V, 80V, 90V	Lower
	99G	30V, 50V, 70V, 80V, 90V	Lower
	30V	95V, 97V, 99V	Higher
	50V	95V, 97V, 99V	Higher
	70V	90V, 95V, 97V, 99V	Higher
	80V	95V, 97V, 99V	Higher
	90V	97V, 99V	Higher
Drosophila hydei	50G	90V	Lower
	97G	90V	Lower
	30V	90V	Lower
	50V	90V	Lower
	70V	90V	Lower
Calliphora vomitoria	70G	95G, 99G	Higher
	70G	95V, 99V	Higher
	70V	95G, 99G	Higher
	70V	95V, 99V	Higher

Table S3.

Pairwise comparisons for treatments with significant differences in Experiment 2. Treatment 1 is different from Treatment 2, being the mean number of appendages lost higher/lower in Treatment 2. Number = ethanol concentration, W = Walking transport, R= Running transport, PN = PostNord transport.

Species	Treatment 1	Treatment 2	Lower / Higher
Macrolophus pygameus	70W	70R, 95R	Higher
	70W	70PN, 95PN	Higher
	95W	70R, 95R	Higher
	95W	70R, 95R	Higher
	70R	95R	Higher
	95R	70PN	Lower
	70PN	95PN	Higher
Aphidoletes aphidimyza	70W	95R	Higher
	70W	95PN	Higher
	95W	95R	Higher
	95W	95PN	Higher
	70R	95R	Higher
	70R	95PN	Higher
	95R	70PN	Lower
	95R	95PN	Lower
	70PN	95PN	Higher

Table S4.

Pairwise comparisons for treatments with significant differences in Experiment 3. Treatment 1 is different from Treatment 2, being the mean number of appendages lost higher/lower in Treatment 2. Number = ethanol concentration, C = Control, D= Drying pre-treatment, F = Freezing pre-treatment.

Species	Treatment 1	Treatment 2	Lower / Higher
Macrolophus pygameus	70D	70C, 95C	Higher
	70D	95D	Higher
	95D	70F, 95F	Higher
Aphidoletes aphidimyza	70C	95C	Higher
	70C	95D	Higher
	70C	95F	Higher
	70D	95C	Higher
	70D	95D	Higher
	70D	95F	Higher
	70F	95C	Higher
	70F	95D	Higher
	70F	95F	Higher
Drosophila hydei	95D	70F	Lower
Calliphora vomitoria	70F	95F	Higher
